# BRCA-Pathway: a structural integration and visualization system of TCGA breast cancer data on KEGG pathways

**DOI:** 10.1186/s12859-018-2016-6

**Published:** 2018-02-19

**Authors:** Inyoung Kim, Saemi Choi, Sun Kim

**Affiliations:** 1 Department of Computer Science and Engineering, Seoul National University, 1 Gwanak-ro, Seoul, 08826 Korea; 2 Interdisciplinary Program in Bioinformatics, Seoul National University, 1 Gwanak-ro, Seoul, 08826 Korea; 3 Bioinformatics Institute, Seoul National University, 1 Gwanak-ro, Seoul, 08826 Korea

**Keywords:** TCGA breast cancer, KEGG-Pathway, Gene expression, Mutation, Copy number variation

## Abstract

**Background:**

Bioinformatics research for finding biological mechanisms can be done by analysis of transcriptome data with pathway based interpretation. Therefore, researchers have tried to develop tools to analyze transcriptome data with pathway based interpretation. Over the years, the amount of omics data has become huge, e.g., TCGA, and the data types to be analyzed have come in many varieties, including mutations, copy number variations, and transcriptome. We also need to consider a complex relationship with regulators of genes, particularly Transcription Factors(TF). However, there has not been a system for pathway based exploration and analysis of TCGA multi-omics data. In this reason, We have developed a web based system BRCA-Pathway to fulfill the need for pathway based analysis of TCGA multi-omics data.

**Results:**

BRCA-Pathway is a structured integration and visual exploration system of TCGA breast cancer data on KEGG pathways. For data integration, a relational database is designed and used to integrate multi-omics data of TCGA-BRCA, KEGG pathway data, Hallmark gene sets, transcription factors, driver genes, and PAM50 subtypes. For data exploration, multi-omics data such as SNV, CNV and gene expression can be visualized simultaneously in KEGG pathway maps, together with transcription factors-target genes (TF-TG) correlation and relationships among cancer driver genes. In addition, ’Pathways summary’ and ’Oncoprint’ with mutual exclusivity sort can be generated dynamically with a request by the user. Data in BRCA-Pathway can be downloaded by REST API for further analysis.

**Conclusions:**

BRCA-Pathway helps researchers navigate omics data towards potentially important genes, regulators, and discover complex patterns involving mutations, CNV, and gene expression data of various patient groups in the biological pathway context. In addition, mutually exclusive genomic alteration patterns in a specific pathway can be generated. BRCA-Pathway can provide an integrative perspective on the breast cancer omics data, which can help researchers discover new insights on the biological mechanisms of breast cancer.

## Background

Transcriptome data measured at the whole genome level requires interpretation at a higher level. For this reason, biological pathway analysis of transcriptome data has become a standard approach, e.g., Pathview [1], Pathway Inspector [2]. However, existing pathway based analysis tools are not powerful enough for the analysis of omics data from cancer, a complex disease that requires integrated analysis of multi-omics data. For example, single nucleotide variation (SNV) and copy number variation (CNV) are frequently measured for cancer research. Thus, it is necessary to integrate and analyze multi-omics datasets of different types together. A number of tools, including TCGA2STAT [3], have been developed for TCGA multi-omics analysis. However, these tools require experienced programming abilities and knowledge about the detailed specification of TCGA data. Meanwhile, there exist web-based multi-omics data analysis services, such as cBioPortal [4], OASIS [5], NetGestalt [6]. These frameworks provide easy access and interpretation of multi-omics data. OASIS provides multi-omics data such as mutation, CNV, and gene expression in selected cancer types and selected oncogenic pathways. However, multi-omics data is presented in a table format, so there is no information about the relationship between genes. NetGestalt provides network-centric view of multi-omics data by adopting visualization on the horizontal dimension to scale up to large networks. In addition, by zooming into a specific gene, multi-omics data of each gene is shown and protein-protein interactions around the selected gene are provided. KeyPathwayMinerWeb provides online multi-omics network enrichment analysis. From user provided gene expression data and an active gene list, maximally connected subnetworks using PPI are provided. These systems display the relationship of genes on the network but do not present multi-omics data on the network. On the contrary, our system, BRCA-Pathway, is designed to represent relationship between genes and corresponding multi-omics data simultaneously on the KEGG pathways. In summary, pathway based multi-omics analysis system is necessary but challenging due to larger sample sizes and higher dimension (multi-omics). For these reasons, we developed BRCA-Pathway, a web-based interactive exploration and visualization system of TCGA breast cancer data on KEGG pathways to provide broad perspective of TCGA breast cancer data. The major features are:

- Multi-omics data such as SNV, CNV, and gene expression can be visualized simultaneously on KEGG pathway maps, together with TF-TG correlation and relationships among cancer driver genes.
- Users can perform comparative analysis of BRCA data, including selection of differentially expressed genes (DEGs) in arbitrary patient groups, mutual exclusivity module (MEMo) summary of genomic alterations (SNV and CNV).
- Data can be downloaded by REpresentational State Transfer Application Programming Interface (REST API).

## Results

### BRCA-Pathway system design

**Fig. 1:**
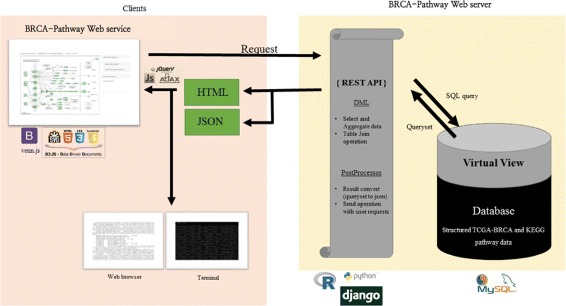
System design for BRCA-Pathway. BRCA-Pathway system structure overview. The left part is client side, and the right part is BRCA-Pathway server side. BRCA-Pathway server builds the database which contains structured TCGA multi-omics data and KEGG pathway data. Database is abstracted by virtual view which simplifies table join functions. REST API could get the data set when it throws query to database. These data set will be provided by Django framework so that the clients can access the data set by web browser or terminal. BRCA-Pathway web front-end receives the data set and visualizes for clients

BRCA-Pathway consists of three components: Database system, REST API, and Web front-end. Overall system design is described in Fig. 1. BRCA-Pathway integrates multiple resources in a relational database and provides a web-based interactive interface and REST API. A database system using MySQL is designed for the structural integration of multi-omics data of TCGA-BRCA, KEGG pathway data, Hallmark gene sets, TF-TG relationships, driver genes, and PAM50 subtype. BRCA-Pathway system can update KEGG Pathways and TCGA-BRCA data by initiating the update software module to incorporate the most recent information. Current configuration is based on KEGG Pathway released on October 1, 2016 and the latest version of GDAC Firehose on January 28, 2016.

### Data sources

BRCA-Pathway stores and utilizes data such as TCGA breast cancer data, breast cancer subtype by PAM50, TF-TG regulation data, Hallmark gene sets, driver gene list and KEGG pathway data. Data is accessible by web-server system or REST API. Data source and status is described in the Table 1.

**Table 1:** Description of data provided by BRCA-Pathway: data type, data size, and data source

Data type	Data size	Data source
Clinical	1098 patients	Standardized analysis-ready TCGA
	22 attributes	data,
	1098 rows	Broad Institute TCGA genome
Expression	1093 patients	data analysis center (2016):
	20,531 genes	Firehose stddata__2016_01_28
	22,440,383 rows	run. Broad Institute of MIT and
Mutation	977 patients	Harvard.
	17,280 genes	doi:10.7908/C11G0KM9
	86,765 rows	
CNV	1080 patients	Broad Institute TCGA genome
	24,776 genes	data analysis center (2016):
	26,758,080 rows	SNP6 copy number analysis (GISTIC2). Broad Institute of MIT and Harvard.doi:10.7908/C1NP23RQ
KEGG pathway	307 pathways	Kyoto encyclopedia of genes and genomes (KEGG)
Transcription factors	85,314 TF-TG pairs	Human transcriptional regulation interaction database
		(HTRIdb)
		Molecular signatures database (MSigDB)
Hallmark gene sets	50 Hallmark gene sets	Molecular signatures database (MSigDB)
Driver genes	486 driver genes	Cancer gene census in COSMIC database
		Vogelstein, Bert, et al. “Cancer genome landscapes.” science
		339.6127 (2013): 1546-1558. Table S2A

### TCGA breast cancer multi-omics data

TCGA breast cancer data was obtained from FIREHOSE [7]. Clinical data was obtained from ‘Clinical_Pick_Tier1’. In addition to clinical data common to all cancer types, breast cancer specific features were selected and stored in BRCA-Pathway system. Additional features are ER, PR, HER2 receptor status and menopause status. Three hormone receptors have been used as the marker of the breast cancer sub-typing [8]. Also, late menopause at age over 55 is known as an risk factor of breast cancer [9]. Gene expression data was obtained from ‘illuminahiseq_rnaseqv2-RSEM_genes_normalized’. Each breast cancer patient has 20,531 gene-level RNA-seq expression data (RSEM normalized counts) from their tumor samples. About 10% of the patient have gene expression data from their normal samples. We used gene expression data from patient wide normal samples as the normal pool data, and gene expression data from patient wide tumor samples as the tumor pool data for the comparison and visualization of gene expression data from selected sub-population. Mutation data was obtained from ‘Mutation_Packager_Oncotated_Calls’. The mutation table includes mutated gene ID, and detailed information about the mutation. Because the patterns of mutation in oncogenes are nonrandom and characteristic, and also because oncogenes are recurrently mutated at the same amino acid positions [10], we included detailed information such as ‘genome_change’ field describing the chromosomal position. CNV data was obtained from ‘CopyNumber Gistic2’.

### Breast cancer subtype data

Breast cancers are classified into subtypes based on gene expression data. PAM50 breast cancer subtyping is widely used method to classify breast cancer into four subtype: Luminal A, Luminal B, Basal-like, HER2-enriched. Breast cancer subtype data is generated using PAM50 predictor bioclassifier R script [11]. The gene expression value of each of PAM50 genes was log2-transformed before the PAM50 run. Users can select the patient group by subtype and TCGA clinical information.

### KEGG pathway data

BRCA-Pathway stored KEGG pathway data by accessing KEGG API [12] and KEGG PATHWAY Database [13]. Gene information such as gene names, KEGG IDs, and relations between pathways and genes was obtained from KEGG REST API [14]. Information about graphical representations of pathway was acquired from KEGG PATHWAY Database [15].

### TF-TG regulation data

KEGG pathway includes information about the gene-gene relation (activation, suppression, gene-gene interaction, etc.). However, relationships between TFs and potential target genes are not provided by the KEGG pathway. To supplement the KEGG pathway, we provide Pearson correlation coefficient between TF-TG with the KEGG pathway. TF-TG relationships were obtained from two different databases. Molecular Signatures Database provides gene sets that share a transcription factor binding site defined in TRANSFAC database [16]. Human Transcriptional Regulation Interaction Database (HTRIdb) is a database for experimentally verified human transcriptional regulation interactions [17]. The union set of both databases are stored in BRCA-Pathway TF-TG table.

### Hallmark gene sets

Hallmark gene sets summarize and represent well-defined biological states or processes and display coherent expression. Fifty Hallmark gene sets were obtained from MSigDB [18]. When researchers are interested in specific biological process, they can start to explore TCGA data by selecting Hallmark gene sets.

### Driver gene list

Driver gene is a gene that contains driver gene mutations or is expressed aberrantly in a fashion that confers a selective growth advantage [10]. We obtained driver gene list consisting of oncogenes and Tumor Suppressor Genes (TSG) from Cancer Gene Census in COSMIC database [19] and the previous research [10]. BRCA-Pathway represents driver genes on the pathway.

### BRCA-Pathway workflow

**Fig. 2:**
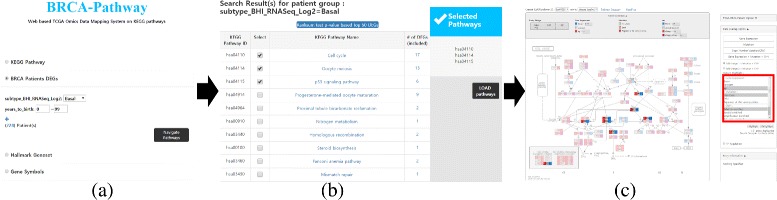
Example workflow of BRCA-Pathway. There are several ways to select KEGG pathways. In this example, pathway selection by DEG is described. First, select subpopulation by breast cancer subtype and clinical features (**a**). If patients with ‘Basal-like’ subtype are selected, then pathways including differentially expressed genes are listed as the result of computation of Wilcoxon rank-sum test (**b**). Select pathways from the list then BRCA-Pathway visualizes multi-omics data of patients whose subtype is ‘Basal-like’. Users can view the pathway by three different data types. Gene expression, mutation, and CNV data are visualized in separate way or integrated way. Genes having specific omics-pattern are highlighted by selecting the omics condition. For example, genes having no mutation and of which CNV is loss or del but overexpressed are selected for highlighting then the pathway turns shaded except the highlighted genes (**c**)

In order to start with BRCA-Pathway, users need to select pathways of their interest. There are several ways to select pathways (Fig. 2). First, users select pathways by differentially expressed genes (DEGs). Users select patient sub-population by PAM50 subtype and clinical information, then BRCA-Pathway starts to perform a Wilcoxon rank-sum test for two groups, i.e., selected sub-population as case and 112 normal samples or remaining rest tumor samples as control. As a result, DEGs and KEGG pathways including DEGs are listed. For example, when users want to explore ‘Basal-like’ subtype patients and select the condition for DEG computation, BRCA-Pathway returns the pathway list including DEGs in ‘Basal-like’ subtype compared with either normal samples or the rest breast tumor samples. After selecting pathways and loading TCGA data, BRCA-Pathway will show gene-expression, CNV and mutation data mapped on selected pathways. Second, users select pathways from Hallmark gene sets. When a hallmark gene set is selected, BRCA-Pathway shows the list of related KEGG pathways by the number of included genes in the selected hallmark gene set. In the same way, users are provided KEGG pathway list by submitting gene symbols.

### Pathway-based exploration of TCGA BRCA

**Fig. 3:**
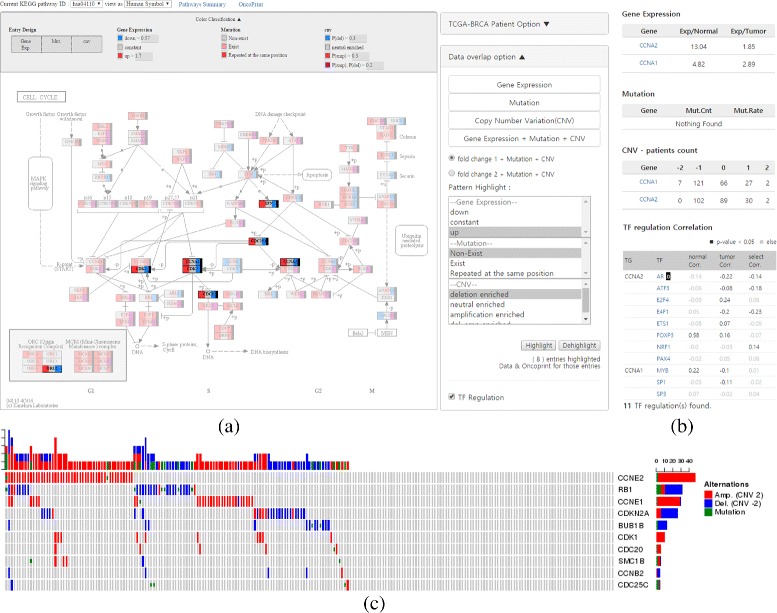
TCGA BRCA multi-omics data exploration with BRCA-Pathway. Visualization of TCGA BRCA with BRCA-Pathway (**a**) Multi-omics data of patients whose subtypes are ‘Basal-like’ is mapped on hsa04110 (Cell cycle). Users can view pathway maps in three different data types. Gene expression, mutation and CNV data are visualized either separately or integrated. Genes with specific patterns are highlighted by selecting the conditions. For example, genes, having no mutation and of which CNV is loss or del but that are over-expressed, are selected. Genes in the pathway except the highlighted genes become shaded (**b**) Clicking a gene entry on the pathway displays multi-omics data of the gene such as gene expression level compared with normal sample pool and with tumor sample pool, mutation count and ratio, CNV as -2 (del), -1 (loss), 0 (neutral), 1 (gain), 2 (amp) with the count of patients having each CNV value and Pearson correlation coefficients of TF-TG gene expression. Three columns represent the correlation of TF-TG in normal sample pool, tumor sample pool and selected subpopulations pool, respectively. Correlation with *p*-value over 0.05 are shaded. **c** Selecting genes, Oncoprint shows copy number variation (amp or del) and mutation in the mutual exclusive way between selected genes. In this case, some extremely over-expressed genes (fold change>10 by normal sample pool) and RB1 are selected and the figure shows mutual exclusive pattern between CCNE2, RB1, CCNE1 and CDKN2A (top 4 rows respectively)

**Fig. 4:**
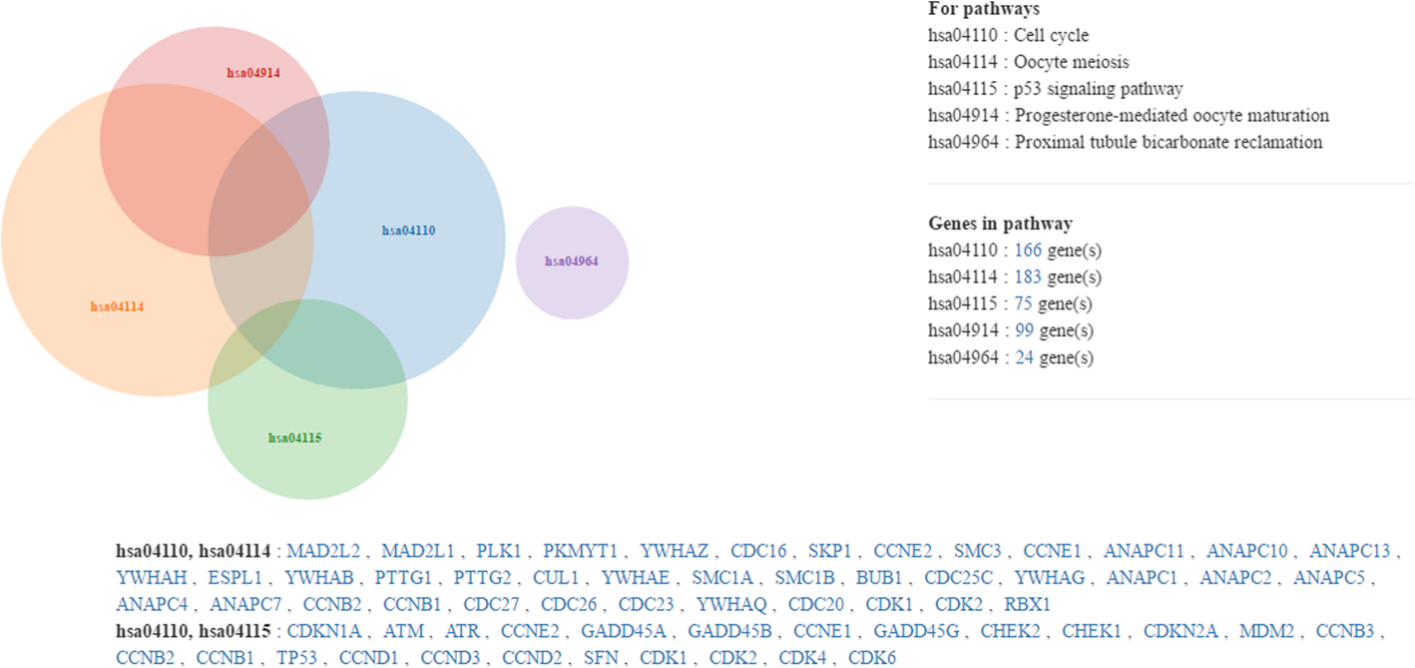
Venn diagram for Pathways summary. Pathway relations are shown in Venn diagram. A list of pathways and the number of genes in each pathway are described on the right side. Genes shared between pathways are listed under the Venn diagram

BRCA data can be explored in different ways. For example, when users specify subpopulation of TCGA breast cancer patients, the system loads multi-omics data of the selected subpopulation into the web-browser. By clicking ‘Data overlap option’, users can change the type of data (gene expression, mutation and CNV) mapped onto the pathway. This enables users to see the same pathway by three different points of view. BRCA-Pathway colorizes KEGG Pathway entries according to the user-controllable classification criteria. The system provides a function that highlights specific patterns of omics data such as gene expression level up, mutation free and copy number deletion (Fig. 3). In addition, ‘Pathways summary’ and ‘Oncoprint’ are available. Pathways summary is shown in Venn diagram [20] that shows overlapped genes among selected multiple pathways (Fig. 4). Genomic alterations such as mutation and CNV in the same pathway are often mutually exclusive [21] and different combination of alterations are sufficient to perturb the pathways [22]. Thus, predicting driver alterations from the frequency of occurrence is not easy. With pathway leveled view, overall trend of genomic alteration is shown, but mutually exclusive patterns of alteration are not recognizable. To compensate this problem, we provide OncoPrint [23] with ‘mutual exclusivity sort’. Users can check on mutual exclusivity in selected patient group with respect to CNV and mutation.

Figure 3a shows a pathway leveled view of ‘Basal-like’ subtype patients and Fig. 3b shows the individual omics data of CCNA1, CCNA2. The highlighted genes are mutation free, copy number loss or deletion enriched but over-expressed compared to normal pool data. Because mutation effects are excluded, there probably exist regulators that promoted the transcription of CCNA1, CCNA2. With provided TF-TG correlation coefficient, users get TF list targeting CCNA1, CCNA2 and see that the correlation coefficients are different between normal samples and selected tumor samples.

### User data visualization on KEGG pathways

BRCA-Pathway provides visualization of user data to extend the usability of the system. After switching to ‘User data mode’, input a text file consisting of Entrez geneId and fold change value, and the gene expression level is shown in color. Adjusting color or threshold value helps to customize pathway visualization. However, unlike ‘TCGA mode’, only a single gene selected by the entry label is considered and all the other genes belonging to the entry are ignored.

### REST API

REST API separates data extraction from the developmental environment so that users can easily extract data without understanding the internal system [24]. Given patient selection option in the web page, a front-end program creates a URL and sends the URL to REST API. This allows the system to aggregate omics data sets for a subset of patients to create a dynamic user view. By using REST API, it is possible to extract genes contained in KEGG pathway maps and to aggregate TCGA data after receiving query result from MySQL. Users can access to data with simple endpoint coding.

The domain address is the server URL that BRCA-Pathway is configured on. After the slash(/) mark, at least one argument should be given. The 1st argument specifies the data to retrieve and the argument can be landscape, search, genes, pathways, TCGA-BRCA. In case of tcga-brca.bhi2.snu.ac.kr/api/landscape, ‘landscape’ represents the current status of TCGA data and KEGG pathway. ‘search’ means that the pathway list will be provided by searching gene names or pathway names, and ‘genes’ provides the gene list in pathways specified by argument 2. Furthermore, ‘pathways’ returns pathway information specified by argument 3’s endpoint filtered by argument 2. REST API examples are listed below. The last example means that it will provide the result of aggregating CNV data from TCGA-BRCA data given the patients option is male and the genes filtered by the pathway ‘hsa00010’. For more customized use, reference Table 2.

**Table 2:** REST API arguments and parameters supported by BRCA-Pathway

arg1	arg2	arg3	Parameters	Description
landscape	-	-	type=[JSON | LIST]	
			fields=[KEGGPathway_update	
			| KEGGPathways	
			| Pathway_Pathway_Membership	KEGG pathways, TCGA-
			| TCGAGenes	BRCA data status
			| TCGA_BRCA_Patients	
			| TCGA_BRCA_update	
			| TFTG_relation | driver_genes]	
search	-	-	keyword	
			type=[ JSON | LIST ]	Get search result by
			fields=[ keyword	keyword.
			| gene_labels	Gene or pathway names
			| rate	with input_type.
			| pathID | pathname ]	
genes	(pathIDs,	-	type=[ JSON | LIST ]	
	sep=“+”)	count	fields=[ pathID	Retrieve pathways
			| gene_symbol	information.
			| pathname	related_pathways operation
			| geneID	supported.
			| count** ]	
			**given only arg3 related_pathways	
pathways	(pathIDs,	-	type=[ JSON | LIST ]	
	sep=“+”)	related_pathways	fields=[ pathID | pathname	Retrieve pathways
			| category_level2_id	information.
			| description	related_pathways operation
			| related_pathID**]	supported.
			**given only arg3 related_pathways	
TCGA-BRCA	patient_list	-	type=[ JSON | LIST ]	
	patient_count	-	** arg2 patient_list	
	(pathIDs,	(given pathIDs,)	fields=[patient_barcode]	Retrieve pathways and
	sep=“+”)	fold_change		TCGA-BRCA omics data.
		mutation	** arg2 patient_count	Gene expression with
		CNV	fields=[count]	fold_change value,
				mutation with the number
			** arg3 fold_change	of patients having
			fields=[geneID |gene_symbol	mutation, and CNV with
			| exp_select	GISTIC2 result range from
			| exp_tumor	-2 to 2.
			| exp_normal	Each omics result will be
			| fc_select_tumor | fc_select_normal ]	given by gene level
				aggregating patients
				group omics data
			** arg3 mutation	patient_list,
			fields=[ geneID | gene_symbol	patient_count operation
			|count ]	supported.
			** arg3 CNV	
			fields=[geneID | gene_symbol	
			| cnv_pos	
			| cnv_zero	
			| cnv_neg ]	
			** And patient_options	
			described_above

- tcga-brca.bhi2.snu.ac.kr/api/landscape
- tcga-brca.bhi2.snu.ac.kr/api/search?keyword=erbb1
- tcga-brca.bhi2.snu.ac.kr/api/genes/hsa00010+hsa00030
- tcga-brca.bhi2.snu.ac.kr/api/pathways/hsa00010/related_pathways
- tcga-brca.bhi2.snu.ac.kr/api/TCGA-BRCA/hsa00010/CNV?gender=male

Patient_options are listed below:

- subtype_BHI_RNASeq_Log2 : all |*Basal*|Her2 |*LumA*|*LumB*|Normal
- years_to_birth_from : integer & years_to_birth_to : integer
- er_status : all |*indeterminate*|*negative*|positive
- pr_status : all |*indeterminate*|*negative*|positive
- her2_status : all |*indeterminate*|*negative*|*positive*|equivocal
- vital_status : all |0|1 *0: alive, 1:dead
- pathologic_stage : all |stage_i |stage_ii |stage_iii |stage_iv |stage_iv |stage_tis |stage_x
- pathologic_T_stage : all |t1 |t2 |t3 |t4 |tx
- pathologic_N_stage : all |n0 |n1 |n2 |n3 |nx
- pathologic_M_stage : all |cm0_ |m0 |m1 |mx
- gender : all |*female*|male
- radiation_therapy : all |*no*|yes
- histological_type : all |infiltrating_carcinoma_nos |infiltrating_ductal_carcinoma |in-filtrating_lobular_carcinoma |medullary_carcinoma |metaplastic_carcinoma |mixed_history(please_specify) |mucinous_carcinoma |other__specify
- number_of_lymph_nodes : all |0|1|2 *1: #of node less than or equal to 10, 2: greater thane 10
- race : all |american_indian_or_alaska_native |*asian*|black_or_african_american |white
- ethnicity : all |hispanic_or_latino |not_hispanic_or_latino
- menopause_status : all |*indeterminate*|*peri*|*post*|pre

## Discussions

### Survival analysis

**Fig. 5:**
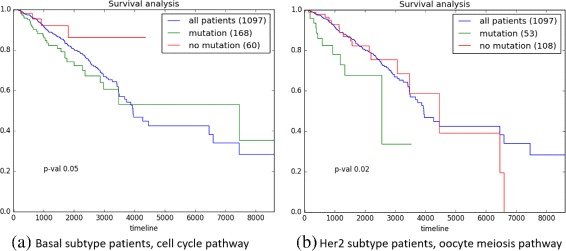
Survival analysis. **a** ‘Basal’ subtype and ‘Cell cycle’ pathway are selected, patients with at least one mutation in the genes involved in the ‘Cell cycle pathway will belong to the mutation group (green line). On the other hand, patients without a mutation in ‘Cell cycle’ genes will belong to the mutation free group (red line). **b** ‘Her2’ subtype and ‘Oocyte meiosis’ pathway are selected, ‘Her2’ patients having at least one mutation in ‘Oocyte meiosis’ genes are depicted as green line, and the rest ‘Her2’ patients are depicted as red line. *P*-value by the logrank test is provided for the comparison of two groups, and all breast cancer patients are depicted as blue line for the convenience

BRCA-Pathway provides survival analysis of selected patients. It divides the selected patients into two groups according to the presence of mutation in genes belong to a particular pathway and provides survival analysis for the patient population. For example, if user selected ‘Basal’ subtype and ‘Cell cycle’ pathway then patients of ‘Basal’ subtype are divided into two groups, mutation group and mutation free group. Patients having at least one mutation in the genes involved in the ‘Cell cycle’ pathway belong to the mutation group. On the other hand, patients without a mutation in ‘Cell cycle’ pathway belong to the mutation free group. Two hundred twenty-eight patients of ‘Basal’ subtype are divided into mutation group (168 patients) and mutation free group (60 patients). The green and red line represent the survival curves of mutation group and mutation free group, respectively. *P*-value by the logrank test is provided for the comparison of two groups, and all breast cancer patients are depicted as blue line for the convenience. Figure 5a gives a *p*-value of 0.05, which indicates a significant difference between the survival curves. Figure 5b gives a *p*-value of 0.02, the survival curve of ‘Her2’ patients having mutation in ‘Oocyte meiosis’ and ‘Her2’ patients having no mutation in ‘Oocyte meiosis’ pathway.

### Gene prioritization

If the user wants to prioritize genes that is significant in ‘Basal’ subtype breast cancer, the exploration can start from DEGs in ‘Basal’ subtype. By selecting patient subpopulation as ‘Basal’ and comparison condition as normal pool, then DEGs and related pathways are shown. Since the ‘Cell cycle’ pathway is listed at the top rank, it is natural to select and load ‘Cell cycle’ pathway for the next step. After loading a pathway, the user can explore multi-omics data on the ‘Cell cycle’ pathway of ‘Basal’ subtype patients. Figure 3a shows a pathway level view of gene expression, mutation and copy number variation. By highlight function, user can locate genes with the specified condition. The highlighted genes are mutation free, copy number loss or deletion enriched but over-expressed compared to normal pool data. Individual multi-omics data is obtained by clicking each entry and Fig. 3b shows the multi-omics data of CCNA1, CCNA2. Because mutation effects are excluded, it would be interesting to see if there exist regulators that promoted the transcription of CCNA1 and CCNA2. With ‘TF regulation’ checked, TF-TG correlation coefficients are provided and then user can get the TFs targeting CCNA1, CCNA2 and see that the correlation coefficient between FOXP3 and CCNA2 is much different in normal samples (0.58) and tumor samples (0.16). Since *p*-value is over 0.05, correlation coefficient in ‘Basal’ subtype samples (-0.07) is shaded. Although we could not identify the responsible genes that promoted transcription of CCNA1 and CCNA2, we found that positive correlation between FOXP3 as TF and CCNA2 as TG is disrupted in tumor samples.

## Conclusions

BRCA-Pathway helps researchers navigate multi-omics data towards potentially important genes, regulators, and discover complex patterns involving mutations, CNV, and gene expression data of various patient groups in the biological pathway context. In addition, mutually exclusive genomic alteration patterns in a specific pathway can be generated. BRCA-Pathway can provide an integrative perspective on the breast cancer omics data, which can help researchers discover new insights on the biological mechanisms of breast cancer. In the future, BRCA-Pathway could include other omics data sets such as miRNA expression and DNA promoter methylation profiles to support more extensive research. And besides breast cancer data of TCGA, other cancer dataset availability is also needed.

## Abbreviations

CNV: Copy number variation
DEG: Differentially expressed genes
HTRIdb: Human transcriptional regulation interaction database
KEGG: Kyoto encyclopedia of genes and genomes
MEMo: Mutual exclusivity module
MSigDB: Molecular signatures database
PAM50: Prediction analysis of microarray 50
REST-API: Representational state transfer application programming interface
RNA: Ribonucleic acid
RNA-seq: Whole transcriptome sequencing
SNV: Single nucleotide variation
TCGA: The cancer genome atlas
TF: Transcription factor
TG: Target gene
TSG: Tumor suppressor gene
